# Exploring the interplay between *Fusobacterium nucleatum* with the expression of microRNA, and inflammatory mediators in colorectal cancer

**DOI:** 10.3389/fmicb.2023.1302719

**Published:** 2023-11-23

**Authors:** Narjess Bostanghadiri, Shabnam Razavi, Aref Shariati, Malihe Talebi, Shiva Mirkalantari, Amirnader Emami Razavi, Davood Darban-Sarokhalil

**Affiliations:** ^1^Department of Microbiology, School of Medicine, Iran University of Medical Sciences, Tehran, Iran; ^2^Microbial Biotechnology Research Center, Iran University of Medical Sciences, Tehran, Iran; ^3^Department of Medical Laboratory Sciences, Khomein University of Medical Sciences, Khomeyn, Iran; ^4^Iran National Tumor Bank, Cancer Institute of Iran, Tehran University of Medical Sciences, Tehran, Iran

**Keywords:** *Fusobacterium nucleatum*, colorectal cancer, cytokines, miR-21, miR-31

## Abstract

**Background:**

*Fusobacterium nucleatum* has been recognized as an important key bacterium in the cause and spread of colorectal carcinogenesis. Nevertheless, the clinical relevance of *F. nucleatum* in colorectal cancer (CRC) and its effect on immune factors and the tumor microenvironment have not been fully elucidated.

**Materials and methods:**

The frequency of *F. nucleatum* was measured in 100 paired tumor and normal tissue specimens by TaqMan quantification Real-Time Polymerase Chain Reaction (qPCR). The mRNA expression levels of cytokines (*IL-6, IL-10, IL-12*β, *IL-17, TNF-*α*, TLR-2*, and *TLR-4*), and miRNAs (*miR-21, miR-31*) were examined. Eventually, any potential correlations between the molecular and clinicopathological features of the neoplastic samples and the abundance of *F. nucleatum* were analyzed.

**Results:**

The relative frequency of *F. nucleatum* was significantly increased in cancerous tissue compared to adjacent non-tumor tissues. Furthermore, the high level of *F. nucleatum* was significantly associated with histological grade III and IV CRC tissues (*P* = 0.027 and *P* = 0.022, respectively) and perineural invasion-positive patients (*P* = 0.037). In addition, the expression levels of *IL-6, IL-17, TNF-*α*,IL-12*β*, TLR-2*, and *TLR-4* as well as *miR-21* and *miR-31* showed a significant increase in the cancer group. A notable correlation was also observed between the high status of *F. nucleatum* and the expression of *IL-6, TNF-*α and *miR-21*.

**Conclusion:**

Our results emphasize the importance of *F. nucleatum* and changes in the expression of genes involved in CRC. Studying the microbial profile and gene expression changes in CRC patients may be a promising approach to improve screening methods and provide therapeutic strategies.

## 1 Introduction

Colorectal cancer (CRC) is a major global health problem, ranking second in mortality with more than 600,000 deaths and third in incidence with more than 1.8 million new cases annually arising. The International Agency for Research on Cancer (IARC) has predicted that the global number of mortality and new CRC cases will increase to 3 and 1.6 million, respectively, by 2040 (Cheng et al., 2020; Kaźmierczak-Siedlecka et al., 2020; Sung et al., 2021; Stolzer, 2022). The etiology of CRC includes both genetic and environmental factors. CRC with heritability aspects that account for only 12–35% of the risk factors includes multiple associated polyposis (MAP), hereditary non-polyposis colorectal cancer (HNPCC or Lynch syndrome), multiple associated polyposis (MAP), Peutz-Jeghers syndrome and familial adenomatous polyposis (FAP) (Dekker et al., 2019; Stolzer, 2022). Environmental factors have a significant impact on the occurrence and progression of CRC, including sedentary lifestyle and obesity, smoking, heavy alcohol consumption, diabetes mellitus, and bad eating habits such as consumption of a diet containing low fiber, high fat, and food carcinogens (Kaźmierczak-Siedlecka et al., 2020; Stolzer, 2022). Among the aforementioned environmental factors, intestinal microbiota is a crucial component in colorectal carcinogenesis.

Previous research points toward the importance of the human intestinal microbiota in influencing normal physiological activities that contribute to the maturation of the immune system and act as a barrier to pathogens (Rea et al., 2018; Dadgar-Zankbar et al., 2023). Gut microbiota dysbiosis, described as an increase in the levels of pathogens alongside a decrease in the levels of beneficial bacteria, is caused by a variety of factors including lifestyle and dietary changes, widespread use of antibiotics, chronic stress and host genetics (Chen et al., 2019). Bacterial dysbiosis is closely associated with inflammatory gastrointestinal diseases such as ulcerative colitis, inflammatory bowel disease (IBD) and CRC. They lead to carcinoma by inducing inflammatory responses, boosting inflammation, aberrant immune regulation, activation of tumorigenic pathways, production of oncogenic substances, metabolic dysregulation, and damage to host DNA (Chen et al., 2019; Wong and Yu, 2019).

Current studies have identified several specific bacterial species associated with the initiation and progression of CRC through the aforementioned mechanisms, including *Fusobacterium nucleatum*, *Bacteroides fragilis, Salmonella enterica*, and *Escherichia coli* (Zeller et al., 2014; Yachida et al., 2019). Interestingly, considerable evidence has shown that *F. nucleatum* is more abundant in colorectal tumor tissue compared to adjacent normal mucosa, suggesting its potential involvement in the pathogenesis of CRC (Allali et al., 2015; Thomas et al., 2019; Wong and Yu, 2019; Tran et al., 2022). *F. nucleatum* is an invasive, pro-tumorigenic, and pro-inflammatory pathogen that adheres to and invades epithelial and endothelial cells by means of several virulence factors and induces inflammatory cytokines in the mucosa surrounding the tumor. This interaction is recognized by Toll-like Receptors (TLRs) and leads to activation of both innate and adaptive immunity (Sakamoto and Maeda, 2010; Huang et al., 2014; Brennan and Garrett, 2016; Henstra et al., 2021). Chemokines and cytokines, which are examples of inflammatory mediators, are extensively produced by inflammatory cells and alter the immune system as well as a wide range of cancers. These cytokines, such as IL-6, IL-17, and TNF-α, directly promote tumorigenesis, tumor cell proliferation, angiogenesis, metastasis, and cell death (Bhat et al., 2022). It is noteworthy that inflammatory cells can also produce cytokines that restrict tumor growth, such as IL-10 and IL-12, which lead to modulating apoptosis and suppressing angiogenesis. However, some studies show their dual role as both tumor promoters and inhibitors (Kabel, 2014).

Numerous genetic factors can serve as molecular markers for CRC diagnosis and prognosis. Among these factors, the family of MicroRNAs (miRNAs) has been identified as a promising candidate (Ghafouri-Fard et al., 2021). miRNAs are small, endogenous, non-coding RNA molecules of 18–23 nucleotides. Aberrant expression patterns and functional abnormalities of miRNAs have been observed in inflammatory processes and several types of human cancer. MiRNAs can act as either tumor inhibitors or oncogenes, depending on their downstream target genes (Chi and Zhou, 2016). miR-21 and miR-31 are considered to be critical miRNAs in CRC and exhibit a statistically significant increase in expression levels in CRC patients compared to the healthy group (Eslamizadeh et al., 2018). miR-21 is a highly prominent miRNAs that is involved in cell proliferation and invasion in CRC via targeting of phosphatase and tensin homolog (PTEN) and Programmed cell death protein 4 (PDCD4) (Li et al., 2014; Shen et al., 2019). Furthermore, miR-31 has been identified as a potent cancer-related miRNA that plays a role in CRC carcinogenesis by targeting tumor suppressor genes (Cottonham et al., 2010).

However, there are few studies regarding the interaction and regulation between the presence of *F. nucleatum* and inflammatory genes and miRNAs in CRC. Therefore, in the current study, we investigated the amount of *F. nucleatum* and its relationship with the expression of inflammatory genes (*TLR-2, TLR-4, TNF-*α*, IL-6, IL-10, IL-12β*, and *IL-17*) and miRNAs (*miR-21*, and *miR-31*) in tumor and adjacent normal tissue of Iranian CRC patients.

## 2 Materials and methods

### 2.1 Sample preparation

A total of 100 pairs of fresh-frozen colorectal adenocarcinoma and matched adjacent non-tumor tissues were provided by the Iran National Tumor Bank, which was founded by the Cancer Institute of Tehran University of Medical Sciences, for Cancer Research, during the 2021–2023 period. After the surgical procedure, the tissue samples were expeditiously conveyed to the pathology unit for expert inspection and assessment by a pathologist. In addition, a segment of the tumor tissues as well as normal adjacent mucosal samples were selected and fixed in RNAlater stabilization buffer (QIAGEN, Hilden, Germany). Samples were preserved at −70°C for further analysis. All clinical histopathological parameters and necessary information were captured from patients’ records. Patients who had colorectal tumors of other types than adenocarcinoma or concomitant malignant tumors originating from other organs were excluded from the study. All patients were new cases and did not use any treatment methods such as antibiotics, probiotics, radiotherapy, or chemotherapy before surgery.

### 2.2 Acid nucleic extraction and cDNA reverse transcription

Total DNA and RNA were extracted from the frozen CRC and adjacent normal mucosal tissue specimens using a FavorPrepTM DNA Mini Kit and FavorPrep™ Tissue Total RNA Mini Kit (Favorgen, Taiwan, Cat. No: FATGK 001), respectively, according to the manufacturer’s instructions. A nanodrop instrument (WPA Biowave II Nanospectrophotometer, USA) at OD 260 and 280 nm was used to measure the concentration and purity of the extracts. A reverse transcriptase reaction was performed using the cDNA synthesis kit (Yekta Tajhiz Azma, Iran and Cat. No: YT4500). For further analysis, the DNA and synthesized cDNA were stored at −20°C.

### 2.3 Relative quantification of *F. nucleatum*

The levels of *F. nucleatum* in both cancerous and matched normal tissues were identified by employing the 16S rDNA gene sequence through the utilization of a real-time TaqMan primer/probe on a Rotor-Gene 6000 real-time PCR cycler (Qiagen Corbett, Hilden, Germany). The gene solute carrier organic anion transporter family member 2A1 (SLCO2A1) of the human reference was used to normalize the Cycle quantification (Cq) values, as previously elucidated (Mima et al., 2016). The primer and probe sequences are indicated in Table 1, and their specificities were examined through the use of EMBL-EPI, NCBI BLAST databases, and Allele ID software (v.7.5). Each reaction mixture, having a total volume of 20 μl, contained 20 ng of genomic DNA, 0.5 μM of each primer, 0.25 μM of the probe, 9 μl of Universal Probe Ex Taq PCR Master Mix (Ampliqon, Denmark), and deionized distilled water (6 μL). The qPCR experiment was conducted as follows: an initial incubation at 95°C for 15 min, followed by 40 cycles of denaturation at 95°C for 15 s, and annealing/extension at 62°C for 30 s. Moreover, all assays for each individual sample were carried out in duplicate in a single patch, and the average outcomes of the dual qPCR investigations were utilized for statistical assessment. The negative control in all analyses consisted of all the components of the reaction mixture, excluding bacterial genomic DNA. Following the guidelines on the minimum information for publication of quantitative real-time PCR experiments (MIQE) (Bustin et al., 2009) (Supplementary material). In addition, *F. nucleatum* ATCC 25586 was used as a positive control. The computation of the fold change (2^–ΔΔCq^) in *F. nucleatum* abundance in tumor compared to normal tissue involved subtracting ΔCq tumor from ΔCq normal, where ΔCq represents the average Cq value difference between each *F. nucleatum* and the reference gene (Mima et al., 2016).

**TABLE 1:** Utilized oligonucleotide primers and TaqMan probes in the present study.

Target	Primer/ probe	Oligonucleotide sequence (5′_3′)	Size (bp)	Product size (bp)	References
*Fusobacterium nucleatum*	Primer F	AGCTACAAGAGAAGAAAATGAAAATGG	27	105	Shariati et al., 2021
	Primer R	CCAACTCCTACAAATCCAGTAACC	24		
	Probe	TTACTTCATACCATACACGAGGATCTACTT	30		
*SLCO2A1*	Primer F	GAGAGATTTGAATGTTGGACAAAGC	25	89	Shariati et al., 2021
	Primer R	ACACTTCTGTGGTCACTCGTC	21		
	Probe	TCCTACTGCCATCCTTCTACCTGCCA	26		
*IL-6*	Primer F	ACTCACCTCTTCAGAACGAATTG	23	149	Wang et al., 2021
	Primer R	CCATCTTTGGAAGGTTCAGGTTG	23		
*IL-10*	Primer F	GTAGATGCCTTTCTCTTGGAGC	24	160	Zonoobi et al., 2018
	Primer R	CCCAGACATCAAGGCGCATGTG	22		
*IL-12β*	Primer F	GCTTCTTCATCAGGGACATCATC	23	112	Bidkhori et al., 2020
	Primer R	CTCCAGGTCTCAGGGTACTC	20		
*IL-17*	Primer F	CAGCAAGAGATCCTGGTCCTG	21	176	Zonoobi et al., 2018
	Primer R	GGTCGGCTCTCCATAGTCTAAC	22		
*TNF-*α	Primer F	CCCCAGGGACCTCTCTCTAATC	22	98	Leija-Martínez et al., 2021
	Primer R	GGTTTGCTACAACATGGGCTACA	25		
*TLR-2*	Primer F	ATCCTCCAATCAGGCTTCTCT	21	163	Shan et al., 2011
	Primer R	ACACCTCTGTAGGTCACTGTTG	22		
*TLR-4*	Primer F	TTGAGCAGGTCTAGGGTGATTGAAC	25	143	Shan et al., 2011
	Primer R	ATGCGGACACACACACTTTCAAATA	25		

IL: interleukin; TLR: toll-like receptor; TNFα: tumor necrosis factor; *SLCO2A1*: solute carrier organic anion transporter family member 2A1.

### 2.4 Inflammatory and anti-inflammatory genes expression

In the present study, relative quantification real-time PCR was used to assess the expression of the interleukin *(IL)-6, IL-10, IL-12*β*, IL-17, TNF-*α*, TLR-2*, and *TLR-4* genes. All of the primers utilized in this investigation are presented in Table 1. Real-time PCR was performed using a Rotor-Gene 6000 real-time PCR cycler (Qiagen Corbett, Hilden, Germany) according to the manufacturer’s instructions. A final volume of 12.5 μL was used, consisting of 3 μL cDNA template, 0.5 μM appropriate forward and reverse primers, 5.25 μL Power SYBR Green PCR Master Mix (Bioneer, Korea) and 2.5 μL deionized distilled water. All reactions were performed in duplicate and all experiments had a no-template control. Each amplification protocol included an initial denaturation step of 12 min at 95°C, followed by 40 cycles of 15 s at 95°C, 45 s at 58–61.5°C (depending on the primer temperature), and extension at 72°C for 25 s. The SLCO2A1 was used as an internal control, and mRNA levels were quantified using the 2^–ΔΔCq^ approach as described above.

### 2.5 microRNA extraction and cDNA synthesis

The FavorPrep™ miRNA Isolation Kit (Favorgen, Taiwan) was used to extract microRNA (miRNA) from frozen tissues. The integrity of the miRNAs was checked using a nanospectrophotometer. Stem-loop primers for specific reverse transcription (RT) of miRNAs and Ana microRNA detection kit (AnaCell Co, Iran) were used according to the manufacturer’s protocol. Briefly, a 20 mL RT reaction master mix was prepared with a 10 ng microRNA sample, 4 μL RT buffer (5X), 1 μL dNTP (10 mM), 0.5 μL RT enzyme (20 U/mL), and 1.5 μL stem-loop RT primers (5×). The reaction conditions were: 37°C for a duration of 60 min, 70°C for 5 min.

### 2.6 miRNA gene expression

Relative quantification real-time PCR was performed using 2X QPCR Master Mix SYBR Green (AnaCell, Iran) to assess the expression of *miR-21*, and *miR-31* in accordance with the guidelines provided by the manufacturer. Cycle conditions for the aforementioned genes were as follows: initial denaturation at 95°C for 5 min following 40 cycles at 95°C for 30 s, annealing at 60°C for 30 s, and extension at 72°C for 30 s. The mRNA expression levels were analyzed using the 2^–ΔΔ*Cq*^ method as previously described. The relative levels of miR-21 and miR-31 were compared to the geometric mean of U6 snRNA (RNU6B) expression (Schee et al., 2012).

### 2.7 Statistical analyses

Statistical analysis was performed with SPSS version v.26.0 software(SPSS Inc., Chicago, IL, USA) and GraphPad Prism v.8.3.0. A two-tailed *P*-value < 0.05 was considered statistically significant. Paired samples *t*-test was used to compare the relative amounts of *F. nucleatum* and the expression of microRNA and pro-inflammatory genes in the tumor and adjacent normal mucosa of paired samples, while the difference in copy number was analyzed using the rank sum test. Fisher’s exact test and chi-squared test (χ2) were used to assess the relationships between *F. nucleatum* status and clinicopathological and molecular features. Multivariate logistic regression analysis was performed to estimate odds ratios (ORs) and corresponding 95% confidence intervals (CIs) for associations between high *F. nucleatum* DNA levels and clinicopathological features.

## 3 Results

### 3.1 Clinicopathological characterization of CRC patients

The histopathological and demographic features of the individuals are summarized in Table 2. In brief, the study consisted of a total of 59 men and 41 women with a mean age of 56.39 years (SD ± 14.80). The majority of CRC patients exhibited signs of grade-II cancer (36%), characterized as moderately differentiated. while 28%, 17%, 15%, and 4% of patients demonstrated grade III (poorly differentiated), grade IV (undifferentiated), grade I (well-differentiated), and grade X (unknown) cancers, respectively. Based on the initial examinations conducted by a gastroenterologist and the microscopic examinations performed by a pathologist, the involvement frequency of various sections of the intestine in CRC has been recorded in Table 2. Tumor location mainly involved rectum (35%), cecum (15%), and sigmoid colon (13%). Overall, 82 colorectal carcinoma patients were diagnosed with adenocarcinomas, twelve patients had mucinous (colloid) adenocarcinoma, two patients had Signet-ring Cell Adenocarcinoma and one patient had mucinous carcinoid. Notably, around 3% of CRC patients had other histology of CRC.

**TABLE 2:** Correlations between *F. nucleatum* status and clinicopathological features.

No. (%)						
		**Amount of *F. nucleatum* in colorectal carcinoma tissue**				
**Characteristic**		**All patients** **(*n* = 100)**	**High** **(*n* = 28)**	**Low** **(*n* = 16)**	**Negative** **(*n* = 56)**	***P*-value**
Age, mean (SD), year		56.39 (14.80)	56.32 (16.83)	58.31 (11.88)	55.88 (14.69)	0.847
Sex	Female	41(41)	12 (42.90)	6 (37.50)	23 (41.10)	0.941
	Male	59 (59)	16 (57.10)	10 (62.50)	33 (58.90)	
Tumor location	Cecum	15	5 (17.90)	4 (25.00)	6 (10.70)	0.886
	Ascending colon	7	3 (10.70)	0 (0.00)	4 (7.10)	
	Hepatic flexure	2	0 (0.00)	0 (0.00)	2 (3.60)	
	Transverse colon	4	0 (0.00)	0 (0.00)	4 (7.10)	
	Splenic flexure	2	0 (0.00)	0 (0.00)	2 (3.60)	
	Descending colon	3	1 (3.60)	1 (6.30)	1 (1.80)	
	Sigmoid colon	13	3 (10.70)	2 (12.50)	8 (14.30)	
	Rectosigmoid	9	2 (7.10)	1 (6.30)	6 (10.70)	
	Rectum	35	11 (39.30)	5 (31.30)	19 (33.90)	
	Colon, NOS	10	3 (10.70)	3 (18.80)	4 (7.10)	
Tumor size	Size ≤ 5	38 (38.0)	11 (39.30)	6 (37.50)	21 (37.50)	0.986
	Size > 5	62 (62.0)	17 (60.70)	10 (62.50)	35 (62.50)	
Histology	Adenocarcinoma	82	23 (82.10)	14 (87.50)	45 (80.40)	0.597
	Mucinous carcinoid	1	0 (0.00)	1 (6.30)	0 (0.00)	
	Mucinous (colloid) adenocarcinoma	12	4 (14.30)	1 (6.30)	7 (12.50)	
	Signet-ring cell adenocarcinoma	2	1 (3.60)	0 (0.00)	1 (1.80)	
	Other	3	0 (0.00)	0 (0.00)	3 (5.40)	
Histology grade	I (well differentiated)	15 (15.0)	2 (7.14)	1 (6.30)	8 (14.30)	<0.001*
	II (moderately differentiated)	36 (36.0)	3 (10.71)	6 (37.50)	23 (41.10)	
	III (poorly differentiated)	28 (28.0)	12 (42.85)	6 (37.50)	15 (26.80)	
	IV (undifferentiated)	17 (17.0)	11 (39.28)	3 (18.80)	7 (12.50)	
	X (unknown)	4 (4.0)	0 (0.00)	0 (0.00)	3 (5.40)	
Lymphatic invasion	Yes, Nos	58	15 (53.60)	13 (81.30)	30 (53.60)	0.297
	Yes, extensive	1	0 (0.00)	0 (0.00)	1 (1.80)	
	No	38	11 (39.30)	3 (18.80)	24 (42.90)	
	Unknown	3	2 (7.10)	0 (0.00)	1 (1.80)	
Vascular invasion	Yes	65	17 (60.70)	13 (81.30)	35 (62.50)	0.327
	No	35	11 (39.30)	3 (18.80)	21 (37.50)	
Perineural invasion	Yes	38	27 (96.42)	1 (3.57)	10 (17.85)	0.033*
	No	62	1 (3.57)	16 (93.75)	46 (82.14)	
Perineal invasion	Yes	2	0 (0.00)	0 (0.00)	2 (3.60)	0.683
	No	98	28 (100.00)	16 (100.00)	54 (96.40)	
Extramural blood vessel invasion	Yes	7	4 (14.30)	0 (0.00)	3 (5.40)	0.249
	No	93	24 (85.70)	16 (100.00)	53 (94.60)	
Extranodal extension	Yes	9	1 (3.60)	4 (25.00)	4 (7.10)	0.071
	No	91	27 (96.40)	12 (75.00)	52 (92.90)	
Perforation	Yes	6	3 (10.70)	0 (0.00)	3 (5.40)	0.440
	No	94	25 (89.30)	16 (100.00)	53 (94.60)	
Peritoneal seeding	Yes	10	1 (3.60)	3 (18.80)	6 (10.70)	0.215
	No	90	27 (96.40)	13 (81.30)	50 (89.30)	
Pathological T	Tx	2	0 (0.00)	0 (0.00)	2 (3.60)	0.580
	T2	10	4 (14.30)	2 (12.50)	4 (7.10)	
	T3	78	21 (75.00)	11 (68.80)	46 (82.10)	
	T4	9	3 (10.70)	3 (18.80)	3 (5.40)	
	N/A	1	0 (0.00)	0 (0.00)	1 (1.80)	
Pathological N	Nx	1	0 (0.00)	0 (0.00)	1 (1.80)	0.966
	N0	39	11 (39.30)	8 (50.00)	20 (35.70)	
	N1	31	10 (35.70)	4 (25.00)	17 (30.40)	
	N2	28	7 (25.00)	4 (25.00)	17 (30.40)	
	NA	1	0 (0.00)	0 (0.00)	1 (1.80)	
Clinical metastasis	M0	89	25 (90.0)	14 (87.5)	50 (89.40)	0.782
	M1	11	3 (10.70)	2 (12.50)	6 (10.70)	
Stage	Stage I	6	2 (7.10)	1 (6.30)	3 (5.40)	0.957
	Stage IIA	26	7 (25.00)	5 (31.30)	14 (25.00)	
	Stage IIB	2	0 (0.00)	1 (6.30)	1 (1.80)	
	Stage IIIA	4	2 (7.10)	1 (6.30)	1 (1.80)	
	Stage IIIB	26	8 (28.60)	3 (18.80)	15 (26.80)	
	Stage IIIC	25	6 (21.40)	3 (18.80)	16 (28.60)	
	Stage IV	11	3 (10.70)	2 (12.50)	6 (10.70)	
Family history	Yes	33	11 (39.30)	5 (31.30)	17 (30.40)	0.705
	No	67	17 (60.70)	11 (68.80)	39 (69.60)	
Alcohol consumption	None drinker	94	26 (92.90)	15 (93.80)	53 (94.60)	1.00
	Social drinker	6	2 (7.10)	1 (6.30)	3 (5.40)	
Smoking status	Non-smoker	82	26 (92.90)	10 (62.50)	46 (82.10)	0.143
	DX-smoker at diagnosis but discontinued	5	0 (0.00)	2 (12.50)	3 (5.40)	
	Smoker	11	2 (7.10)	4 (25.00)	5 (8.90)	
	Ex-smoker	2	0 (0.00)	0 (0.00)	2 (3.60)	

*: Statistically significant correlation.

### 3.2 *Fusobacterium nucleatum* quantification

**FIGURE 1:**
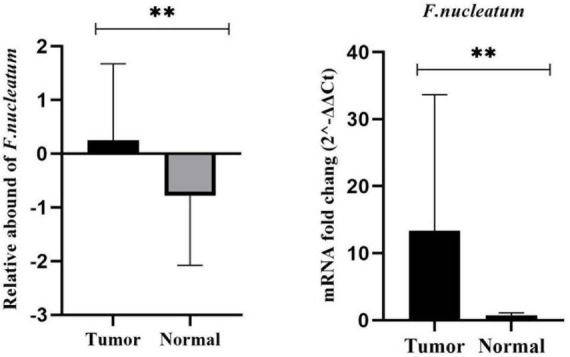
Comparison of the presence of *F. nucleatum* in cancerous and matched normal tissues (*P* < 0.004**).

In this particular research, we quantitated CRC-associated *F. nucleatum* in both colorectal carcinoma tissue and matched normal mucosal samples through relative quantification Real-time-PCR assay. The mean abundance of *F. nucleatum* was significantly higher in CRC tissues in comparison to adjacent normal tissues (CRC vs. normal: 28.57 ± 22.82 vs 6.04 ± 10.45, *n* = 100, *P* = 0.004, paired *t*-test) (Figure 1). *F. nucleatum* was detected in 44% and 25% of cancer tissues and adjacent non-tumor tissue, respectively. The Receiver Operating Characteristic (ROC) curve was utilized to calculate an optimal cutoff value, based on the quantity of *F. nucleatum*, to categorize *F. nucleatum*-positive CRCs into low and high groups. Consequently, out of the 44 colorectal carcinoma cases with detectable *F. nucleatum*, 28 cases were split into high-level groups and 16 cases into low-level groups. None of the patients in the high-level group had previous cancer, with a mean age and tumor sample size of 56.32 years (SD ± 16.83) and 6.13 cm (SD ± 2.15), respectively. However, the statistical analyses revealed no significant correlation (*p* > 0.05) between *F. nucleatum* and the aforementioned markers.

### 3.3 Inflammatory and anti-inflammatory gene expression

**FIGURE 2:**
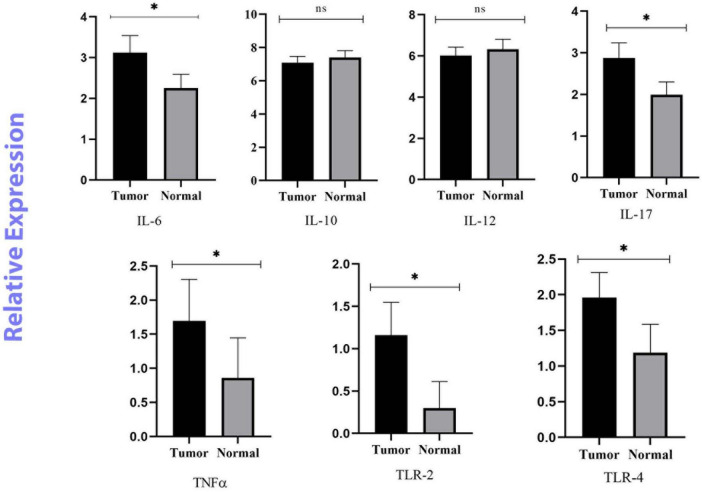
Relative quantification of Inflammatory and anti-inflammatory genes in cancerous and matched normal tissues (*P* < 0.05* and *P* > 0.05^ns^).

In this study, the expression levels of *IL-6, IL-10, IL-12*β*, IL-17, TNF-*α*, TLR-2*, and *TLR-4* were investigated by real-time PCR and 2^–ΔΔCq^. The ROC curve was used to classify the expression of each gene in CRC into low and high groups. *IL-6, IL-17, TNF-*α*, TLR-2*, and *TLR-4* were significantly more highly expressed in the cancer tissues (*p* < 0.05). However, there was no meaningful difference between the tumor and normal samples for *IL-10, and IL-12*β (*p* > 0.05) (Figure 2).

### 3.4 miRNA gene expression

**FIGURE 3:**
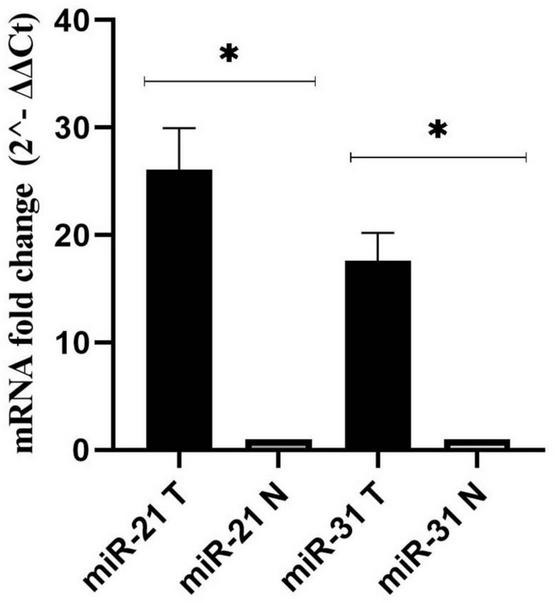
Fold change analysis of *miR-21*, and *miR-31* gene expression in cancerous and matched normal tissues (*P* < 0.001*).

As a control, the U6 gene was used to evaluate the expression levels of *miR-21*, and *miR-31* genes in cancer and adjacent normal tissues (Schee et al., 2012). According to the results, *miR-21* and *miR-31* gene expression was significantly higher in the cancer tissues compared to the adjacent non-tumor mucosa (*P* < 0.001) (Figure 3).

### 3.5 Clinicopathological and molecular association of *F. nucleatum* in CRC

The clinicopathological and molecular characteristics of CRCs according to *F. nucleatum* status (high expression group vs. low expression group vs. negative group) in CRC tissue are presented in Tables 2, 3.

**TABLE 3:** Correlations between *F*. *nucleatum* status and molecular features.

No. (%)						
		**Amount of *F. nucleatum* in colorectal carcinoma tissue**				
**Gene**		**All patients** **(*n* = 100)**	**High** **(*n* = 28)**	**Low** **(*n* = 16)**	**Negative** **(*n* = 56)**	***P*-value**
IL6.T	High	54	27 (96.42)	11 (68.80)	16 (28.57)	0.013*
	Low	46	1 (3.57)	5 (31.30)	28 (50.00)	
IL10.T	High	36	12 (42.90)	5 (31.30)	19 (33.90)	0.660
	Low	64	16 (57.10)	11 (68.80)	37 (66.10)	
IL12.T	High	48	8 (28.60)	8 (50.00)	32 (57.10)	0.590
	Low	52	20 (71.40)	8 (50.00)	24 (42.90)	
IL17.T	High	48	16 (57.10)	6 (37.50)	26 (46.40)	0.427
	Low	52	12 (42.90)	10 (62.50)	30 (53.60)	
TLR2.T	High	45	8 (28.60)	8 (50.00)	29 (51.80)	0.119
	Low	55	20 (71.40)	8 (50.00)	27 (48.20)	
TLR4.T	High	44	10 (35.70)	5 (31.30)	29 (51.80)	0.201
	Low	56	18 (64.30)	11 (68.80)	27 (48.20)	
TNFα.T	High	52	15 (53.60)	10 (62.50)	27 (48.20)	0.047*
	Low	48	13 (46.40)	6 (37.50)	29 (51.80)	
microRNA-21 T	High	82	28 (100.00)	10 (62.5)	44 (78.57)	<0.001*
	Low	18	0 (0.00)	6 (37.50)	12 (22.22)	
microRNA-31 T	High	59	20 (71.42)	6 (37.5)	33 (58.92)	0.063
	Low	41	8 (28.57)	10 (62.5)	23 (41.07)	

IL: interleukin; TLR: toll-like receptor; TNFα: tumor necrosis factor; T: tumor. *: Statistically significant correlation.

According to logistic regression analysis, a significant correlation was observed between high levels of *F. nucleatum* and histology grade III and IV CRC tissues (*P* = 0.027 and *P* = 0.022, respectively), in which the levels of *F. nucleatum* DNA were 5.25-fold and 14-fold higher in grade III and IV CRC tissues, respectively, compared with grade I tissues. Moreover, perineural invasion-positive patients displayed a significant association with high-level *F. nucleatum* (*P* = 0.037). Compared to perineural invasion-negative patients, the presence of *F. nucleatum* DNA was 2.79-fold higher in perineural invasion-positive patients. No significant correlation between *F. nucleatum* infection with other clinicopathological variables was observed (*P* > 0.05).

According to the results, significant positive correlations were observed between the greater amount of *F. nucleatum* and high levels of *IL-6* (*P* = 0.014) and *TNF-*α (*P* = 0.047) genes. Logistic regression results suggested that the level of *F. nucleatum* DNA was 3-fold and 4.4-fold higher in *IL-6* and *TNF-*α high groups compared to low groups. There was a conspicuous correlation between *miR-21* gene expression and *F. nucleatum*-high status in CRC tissues (*P* = 0.012). Conversely, no significant relation was found between the expression of the *miR-31* gene and this group (*P* = 0.063).

## 4 Discussion

The dynamic and observable effect of microorganisms involved in carcinogenesis is a key issue in investigating their role in many types of cancer (Collins and Altman, 2012; Guo et al., 2021). *F.nucleatum*, has been recognized as one of the predisposing factors for CRC and plays a role in the progression of cancer through multiple mechanisms (McIlvanna et al., 2021). Recently, research into the *F. nucleatum* and its relationship with CRC has been the focus of many studies. However, the mechanism of action and the relationship between *F. nucleatum* and other microenvironmental factors in the development of CRC are unclear. To address this knowledge gap and gain a more thorough comprehension of the existing relationship, in this study, we investigated the frequency and association of *F. nucleatum* with the expression of cytokines and miRNAs involved in tumor tissue samples and non-neoplastic mucosa of Iranian patients.

In our current study, *F. nucleatum* was expressed at significantly higher levels in tumor samples compared to the adjacent normal tissues, which is aligned with previous studies (Castellarin et al., 2012; Viljoen et al., 2015; Rye et al., 2022). The percentage of presence of *F. nucleatum* in this study is also within the average range reported in previous studies from different countries (8.6–87%) (Mima et al., 2015; Baxter et al., 2016; Suehiro et al., 2017; Wong et al., 2017; Tunsjø et al., 2019; Kim et al., 2020; Shariati et al., 2021). This discrepancy is influenced by a multitude of factors such as the utilization of different diagnostic methods, various biological samples, and sample quality (Li et al., 2016; Suehiro et al., 2017; Leung et al., 2019). In addition, Lee et al. (2018) found that the method of tissue fixation may influence the variable results of *F. nucleatum* analysis. Shariati et al. (2021) reached similar conclusions by comparing the frequency of *F. nucleatum* in the colorectal tumor specimens and matched normal tissue by quantitative PCR analysis. They found *F. nucleatum* DNA in 23% of CRC biopsies (Shariati et al., 2021). On the other hand, in some studies in Iran, approximately 70% of patients with CRC were colonized by *F. nucleatum* (Kashani et al., 2020; Bostanshirin et al., 2023). The possibility of an excess rate of *F. nucleatum* in these studies may be due to disparities in technical methodologies employed, including simple PCR, SYBR qPCR, and quantitative PCR. Other factors influencing the inconsistency of reports include the diversity of the gut microbiome within the population, dietary habits, and geographical location. Nishijima et al. (2016) have shown that the gut microbiome of Japanese people is significantly different from that of other populations. Of course, this difference cannot be attributed to differences in geographical location alone (Nishijima et al., 2016). Recent studies have shown that the association between *F. nucleatum* and CRC is higher in Asian populations than in European or American populations (Huang et al., 2018; Janati et al., 2020). This population-level association may be explained by differences in lifestyle and diet in different communities, or by population-level variations in the human gut microbiome. Studies have shown that a conservative diet that includes more vegetables, fruits and fiber is associated with a reduced risk of *F. nucleatum*-positive CRC. On the other hand, higher consumption of fats, red/processed meat increases the risk of the *F. nucleatum*-positive CRC tumor subtype. Therefore, the composition and diversity of the colonic microbiota is influenced by dietary changes, and the balance between beneficial and harmful microbial metabolites is crucial in mediating CRC risk factors (Leng et al., 2018; Zhang et al., 2018). However, these results should be investigated further and different factors should be taken into account.

We also assessed the association of *F. nucleatum* status with clinicopathological features and found that high levels of *F. nucleatum* in tumor tissue were related to poorly differentiated and undifferentiated tumors. Previous studies have shown that there is a significant association between elevated levels of this bacterium and poor tumor differentiation (Sun et al., 2016; de Carvalho et al., 2019). Notably, a significant association has been observed between high levels of *F. nucleatum* and perineural invasion (PNI) positive tumors. However, further studies are needed to prove this association. PNI is the invasion of tumor cells into the perineural space of nerves. PNI is a strong prognostic factor in CRC and is significantly associated with reduced survival and high recurrence rates. Studies have shown a significant improvement in 5-year survival in patients with negative PNI tumors compared with those with positive PNI tumors (Poeschl et al., 2010; Betge and Langner, 2011).

The precise mechanisms by which the gut microbiota influences the development of CRC are not fully understood. However, one of the most encouraging hypotheses is that it occurs via microbe-induced inflammation. In particular, inflammation is a critical and well-known risk factor for the development and progression of CRC (Gerhard Rogler et al., 2010). Dysbiosis of gut bacteria may be the cause of immune dysregulation and pro-inflammatory mediator production (Jm, 2009; Swidsinski et al., 2011). Numerous studies have investigated the potential factors by which *F. nucleatum* contributes to colorectal tumorigenesis (McCoy et al., 2013; Hashemi Goradel et al., 2019). The presence of *F. nucleatum* in the gut promotes the expression of tumor-associated cytokines and an inflammatory response through the action of virulence factors.

*Fusobacterium nucleatum* can activate β-catenin signaling through two pathways. The first pathway is the binding of FadA to E-cadherin which can active the zipper mechanism and transport *F. nucleatum* into cells. The second pathway is the TLR4/P-PAK1 cascade that leads to the initiation of inflammatory responses, followed by the amplification of transcription of NF-κβ genes and pro-inflammatory cytokines. Also, it can intensify inflammatory responses through the binding of the Fap2 factor to Gal-GalNAc (Kostic et al., 2013; Rubinstein et al., 2013). TLR-2 and TLR-4 are mainly involved in recognizing *F. nucleatum* and regulating inflammatory factors induced by this bacterium via Tregs (Jia et al., 2017). Both the observations of Kostic et al. (2013) and Rubinstein et al. (2013) support the role of *F. nucleatum* in stimulating the production of an inflammatory microenvironment, leading to an increase in the oncogenic potential of this microorganism (20, 21). Given the reports of previous studies, we assessed the mRNA expression of mucosal inflammatory cytokines and the association between their expression levels and the abundance of *F. nucleatum* in CRC tissues compared to adjacent normal tissues. Our results showed that *IL-6, IL-17, TNF-*α*, TLR-2* and *TLR-4* genes were overexpressed in tumor tissues compared to adjacent normal tissues. *IL-10* and *IL-12*β genes were downregulated, but the difference was not significant. We also found a significant positive correlation between a high amount of *F. nucleatum* and high levels of *IL-6*, and *TNF-*α expression in the tumor tissues.

IL-6 is a pro-inflammatory cytokine with pro-tumorigenic properties. It regulates multiple signaling pathways including survival, invasion, apoptosis, proliferation, angiogenesis and metastasis. Overexpression of *IL-6* has been well-studied in several malignancies including lung, breast, and colon cancer (Sethi et al., 2012; Heichler et al., 2020; Ke et al., 2020). Consistent with our findings, Akhmaltdinova et al. (2020) and Proença et al. (2018) have shown that *IL-6* levels are significantly elevated in CRC patients. In contrast, two British studies found no association between *IL-6* levels and CRC risk (Heikkilä et al., 2009). The small sample size may be a reason for this conclusion. Another study measured the concentration of IL-6 in serum samples from 208 patients with stage I to IV CRC. Patients with stage III and IV disease had significantly higher serum IL-6 concentrations than those with stage I and II disease (Belluco et al., 2000). TNF-α is another pro-inflammatory cytokine that is produced by tumor or inflammatory cells and is involved in the regulation of a variety of signaling processes. Like IL-6, it is involved in tumor initiation, cell proliferation, promotion of angiogenesis, and metastasis (Lan et al., 2021). Consistent with the study by Akhmaltdinova et al. (2020) our data show that *TNF-α* levels are significantly elevated in all CRC tissues. Previously published results on colorectal adenomas found that a high abundance of *F. nucleatum* was positively correlated with the expression of inflammatory cytokine genes, such as *IL-6* and *TNF-α*, which is similar to our findings (McCoy et al., 2013; Velsko et al., 2015; Martinho et al., 2016; Wei et al., 2016). Despite the positive correlation between *Fusobacterium* species and IL-6, the results were not statistically significant in the study by McCoy et al. (2013). Studies have shown that some markers of inflammatory responses, such as IL-1β, IL-6 and TNF-α, are specific to *F. nucleatum* infection and their expression is enriched in *F. nucleatum*-infected CRCs. However, they were not seen in CRC tissue with other bacteria (Wu et al., 2019). As mentioned above, *F. nucleatum* can shape the inflammatory microenvironment in CRC through multiple mechanisms. It binds to colon epithelium and leads to the activation of NF-κB. Also, it can stimulate IL-6 production by activating both TLR-2 and TLR-4 in bone marrow-derived macrophages (Park et al., 2014). *F. nucleatum* increases the infiltration of inflammatory cells such as dendritic cells, M2 macrophage polarization, and granulocytes (Chaushu et al., 2012; Noh et al., 2016). Natural killer (NK) cells can directly recognize *F. nucleatum* through its surface ligand and secrete TNF-α to exacerbate the expression and secretion of IL*-6* (Chaushu et al., 2012). Consequently, the concentration of circulating IL-6 and TNF-α as gastrointestinal inflammation may be an indicator of promoting CRC development.

IL-17 is another cytokine investigated in this study, which is mainly synthesized and released by Th-17 lymphocytes and contributes to the development of terminal inflammation. In addition, IL-17 is a potent immunomodulator and can promote angiogenesis and tumor growth (Kuen et al., 2020). Our results showed a significant difference in *IL-17* expression levels between CRC patients and similar normal tissues, which seems to be in line with the results of other studies, especially in those with poorly differentiated and well-differentiated tumor tissues (Lin et al., 2015; Feng et al., 2019). Taken together, these results support the role of IL-17 in CRC development and progression. In contrast to our results, Stanilov et al. (2010) found no significant difference in IL-17 levels between plasma samples from CRC patients and healthy subjects. Sample type and method of measurement may explain this difference in results.

IL-12 is an inflammatory cytokine that has been shown to play an anti-tumor and anti-metastatic role *in vivo* in a number of murine models of colon adenocarcinoma. The anti-tumor activity of IL-l2 is mediated by activation of Th1 adaptive immunity and increased interferon production, which has a direct toxic effect on cancer cells (Trinchieri, 2003; Lan et al., 2021). Our results showed that the expression level of the *IL-12*β gene was decreased in cancer tissues, suggesting the anti-tumor activity of IL-12β in CRC patients. The findings presented here align with the investigation conducted by O’Hara et al. (1998) in which the authors observed that patients with CRC have decreased IL-12β production. According to the results, the use of IL-12β as a cancer immunotherapy may be beneficial in controlling tumor growth (Briukhovetska et al., 2021).

IL-10 is a cytokine with bidirectional immunomodulatory properties. The immunosuppressive effect of IL-10 on dendritic cells and macrophages results in attenuated antigen presentation, allowing tumor cells to evade immune surveillance and impair cell maturation and differentiation. IL-10 inhibits NF-κB signaling; therefore it can downregulate the expression of proinflammatory cytokines and act as an antitumor cytokine (Li et al., 2020). Abtahi et al. (2017) showed that the serum level of *IL-10* was significantly lower in CRC patients than in controls. This finding is consistent with our current research. However, they highlighted the association between the expression of this cytokine and the prognosis of CRC patients, and those with a poor prognosis had high levels of *IL-10* (Abtahi et al., 2017). Inconsistent with our results, previous studies have shown overexpression of *IL-10* in CRC tissues compared to normal tissues (Li et al., 2019; Cuellar-Gómez et al., 2022). This variation in *IL-10* levels is contingent upon the onset and progression of CRC and supports the potential ambivalent function of IL-10. Nevertheless, further investigation is imperative in order to comprehend the underlying processes of IL-10 whether it is a tumor-stimulating or inhibitory factor.

Until recently, a number of studies have suggested aberrant expression of miRNAs and their oncogenic or suppressive functions in the initiation and progression of various malignancies such as CRC (Nosho et al., 2016; Rapado-González et al., 2019). The trend of *miR-31*, and *miR-21* expression in our results was significantly upregulated in CRC patients compared to adjacent normal tissues, which is similar to previous researches (Kanaan et al., 2012; Wu et al., 2012; Nosho et al., 2014; Wang et al., 2017; Sabry et al., 2019; Farouk et al., 2020; Nassar et al., 2021; Zhou et al., 2022). Therefore, with this comparison, we can refer to the role of these genes in the development of CRC. However, Wang et al. (2014) showed that the expression of miR-31 was significantly decreased in serum samples from patients with CRC. This may demonstrate the dual and contradictory function of miRNA in different types of tumors. miR-31 may not only promote the growth and progression of malignancies such as pancreatic, cervical, and CRC, but also suppress carcinogenesis and induce apoptosis in cancers such as ovarian and prostate cancer (Laurila and Kallioniemi, 2013; Braga et al., 2017). Studies suggest that the diverse role of this factor in cancer regulation may be due to spatiotemporal specificity, characteristics of adenocarcinoma tissue and target genes, which have significant interaction with the signaling pathway (Loya et al., 2009; Yu et al., 2018). However, further research is required to understand the underlying mechanism. In general, miR-21 and miR-31 have been reported to be valuable diagnostic biomarkers for CRC (Sur et al., 2022). We identified a significant association between high levels of *F. nucleatum* and *miR-21* expression in CRC tissue, which is consistent with previous studies (Nosho et al., 2016; Yang et al., 2017; Bostanshirin et al., 2023). *F. nucleatum* can trigger the TLR4/MYD88 signaling pathway by lipopolysaccharide (LPS). Subsequently, hyperactive NF-κB binds to the miR-21 promoter and upregulates its expression in CRC patients (Yang et al., 2017; Yu et al., 2017). This finding partially supports the role of *F nucleatum* in carcinogenesis through the induction of miR21.

In contrast, no correlation was observed between miR-31 expression and the aforementioned colonization. Similarly, Ito et al. (2015) also showed that there was no significant correlation between *miR-31* expression and *F. nucleatum* status. However, Tang et al. (2023) demonstrated that the upregulation of miR-31 was significantly correlated with the presence of *F. nucleatum* in CRC tissues and resulted in the promotion of tumorigenesis. Furthermore, they reported that miR-31-mediated inhibition of autophagic flux via suppression of syntaxin-12 (STX12) was linked to enhanced intracellular survival of *F. nucleatum* infection (Tang et al., 2023). The investigation of the relationship between *F. nucleatum* and miRNAs expression has been very limited. Understanding this relationship will provide new insights into strategies for cancer control and treatment.

## 5 Conclusion

The results showed that the abundance of *F. nucleatum* was significantly greater in cancerous tissue compared to normal tissue. In addition, a significant association was found between *F. nucleatum* and the expression of *miR-21, IL-6* and *TNF-α*. The current findings provide important insights into the function of *F. nucleatum* and its potential association with increased gene expression in carcinogenesis, thereby playing a critical role in CRC progression and metastasis. The data presented provide ample evidence for the pathogenic role of *F. nucleatum* in CRC, thus opening new avenues for targeting the microbiota to accelerate the prognosis of cancer progression and prevent the development of CRC. Furthermore, due to the effect of inflammatory factors and miRNAs and the effect of *F. nucleatum* on their expression, they may serve as biomarkers for acceptable cancer diagnosis.

## Data availability statement

The raw data supporting the conclusions of this article will be made available by the authors, without undue reservation.

## Ethics statement

The studies involving humans were approved by the Ethics Committee of Iran University of Medical Sciences “IR.IUMS.FMD.REC.1400.569.” To ensure safety, all participants were informed of the aims of this study and written informed consent was obtained prior to their participation. The studies were conducted in accordance with the local legislation and institutional requirements. The participants provided their written informed consent to participate in this study.

## Author contributions

NB: Conceptualization, Data curation, Investigation, Methodology, Resources, Software, Writing – original draft, Writing – review and editing. SR: Project administration, Writing – review and editing. AS: Project administration, Writing – review and editing. MT: Project administration, Writing – review and editing. SM: Project administration, Writing – review and editing. AE: Data curation, Writing – review and editing. DD-S: Data curation, Funding acquisition, Methodology, Project administration, Resources, Software, Supervision, Visualization, Writing – review and editing.
