# Supplementary material for: Exploring the interplay between Fusobacterium nucleatum with the expression of microRNA, and inflammatory mediators in colorectal cancer
Source: Front Microbiol. 2023 Nov 23;14:1302719. doi: 10.3389/fmicb.2023.1302719 (PMC10701916; doi:10.3389/fmicb.2023.1302719)
Supplement: Supplementary file 1 [file Data_Sheet_1.PDF]

Supplementary Material

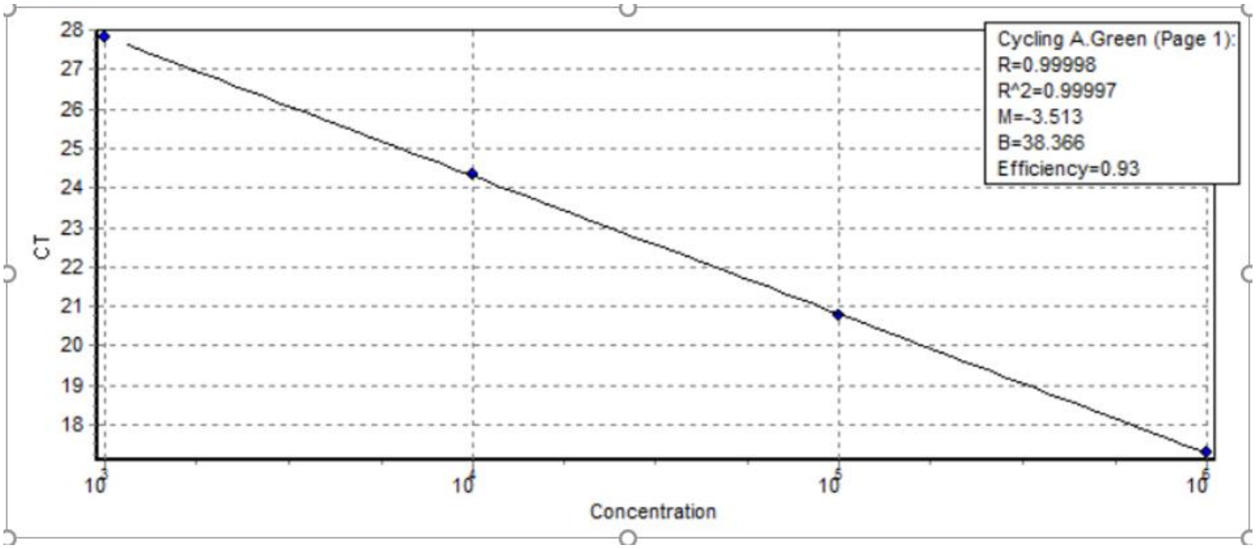

Figure 1: Screen shots from the Rotor-Gene 6000 software of the standard curve for *Fusobacterium nucleatum*

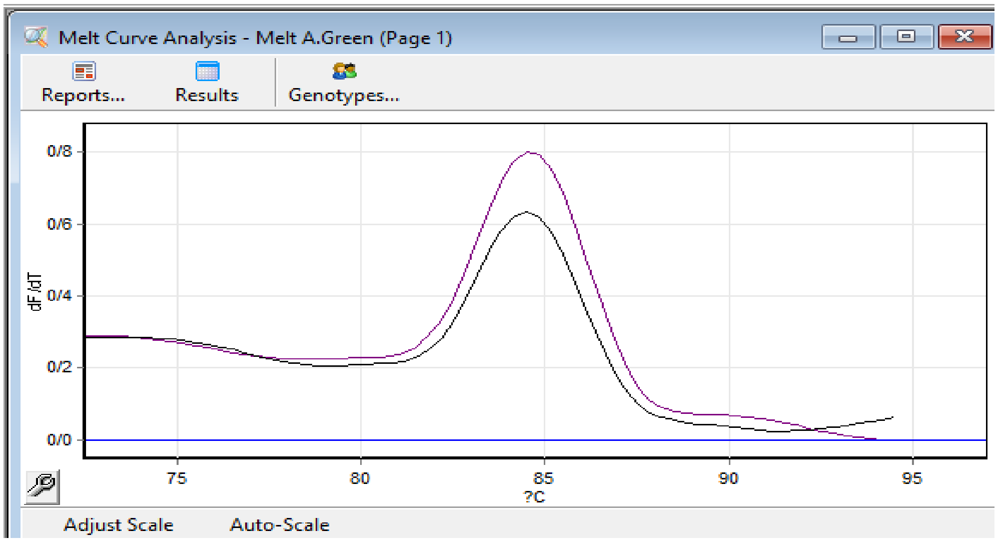

Figure 2: Screen shots from the Rotor-Gene 6000 software of the melting curve for IL-12

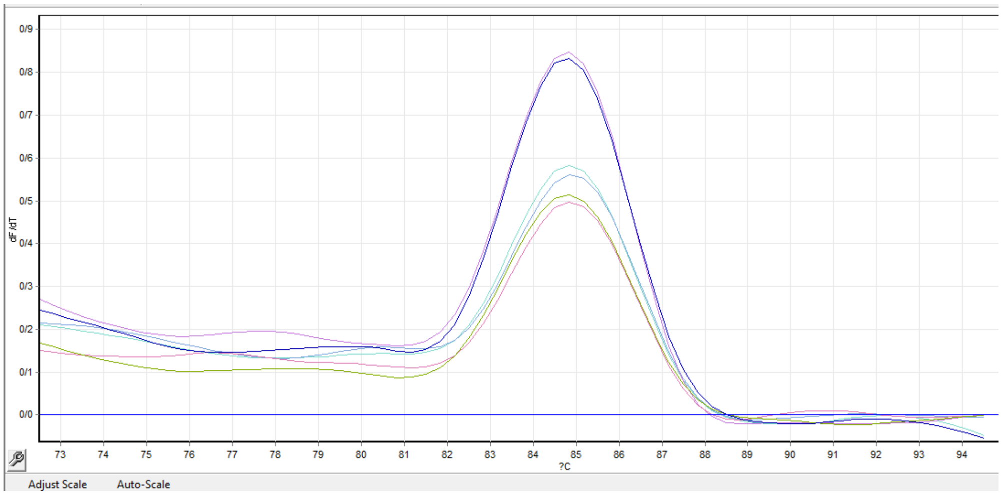

Figure 3: Screen shots from the Rotor-Gene 6000 software of the melting curve for IL-17

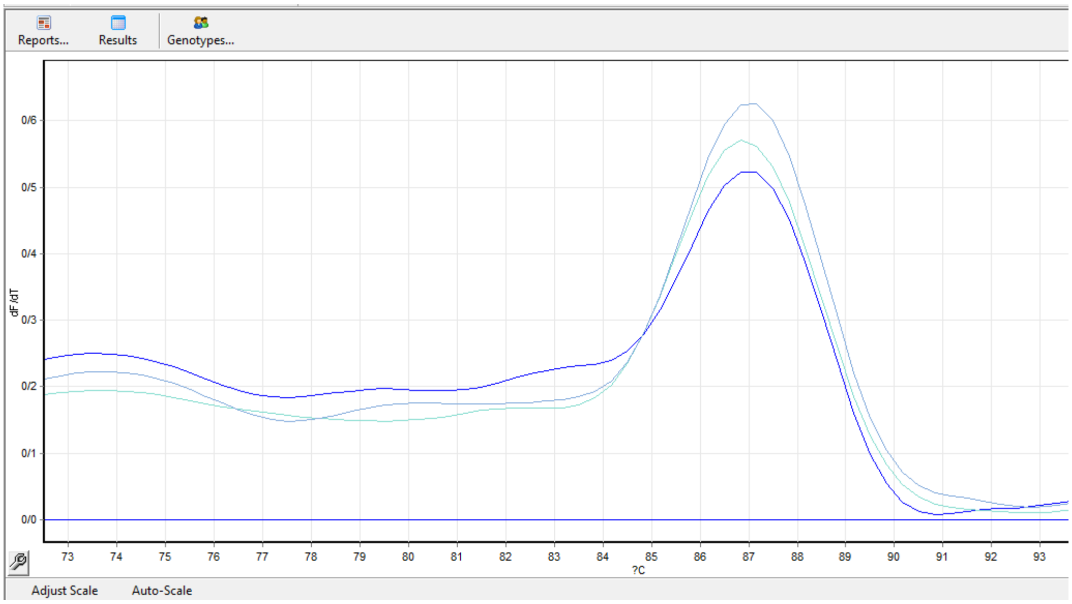

Figure 4: Screen shots from the Rotor-Gene 6000 software of the melting curve for IL-10

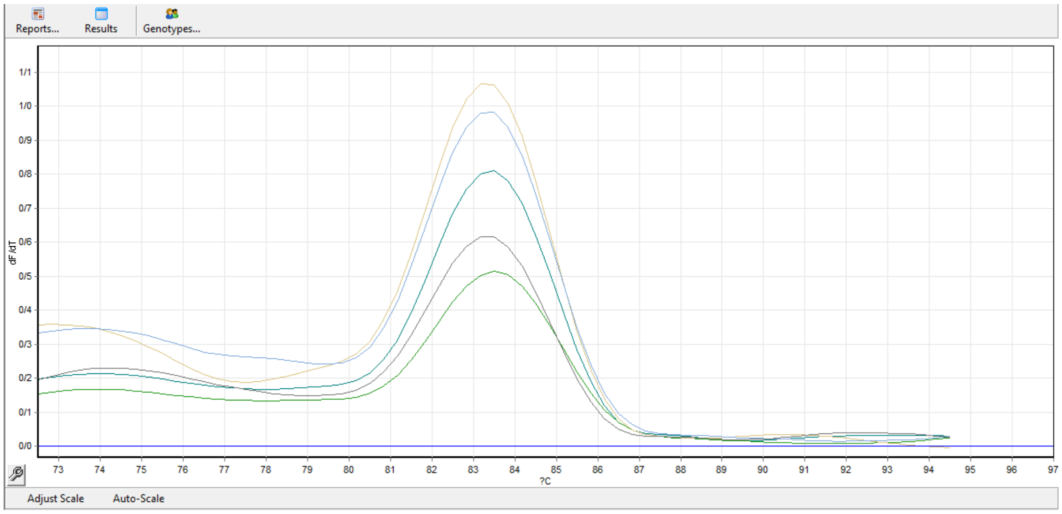

Figure 5: Screen shots from the Rotor-Gene 6000 software of the melting curve for IL-6

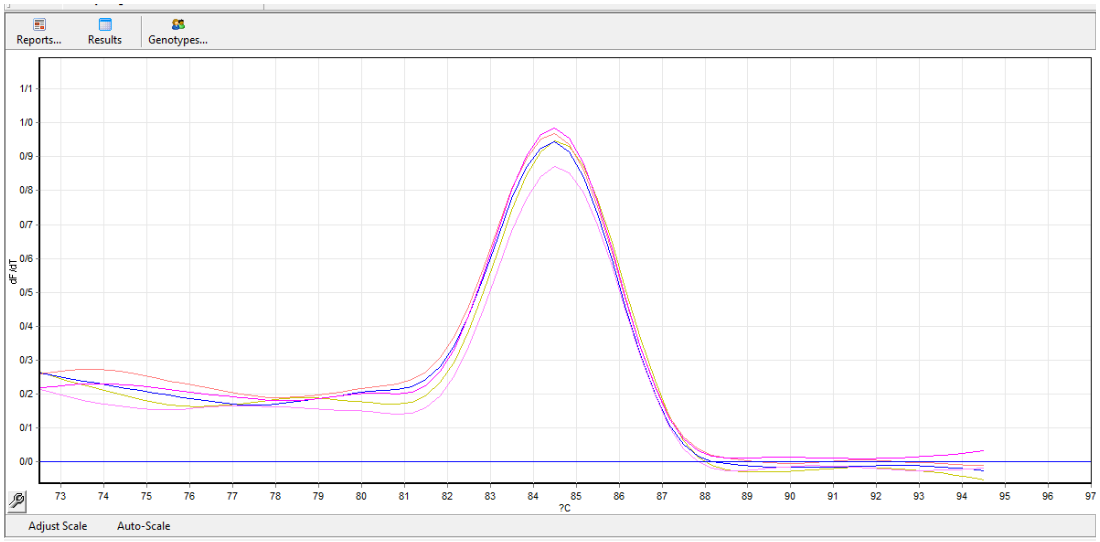

Figure 6: Screen shots from the Rotor-Gene 6000 software of the melting curve for TLR-2

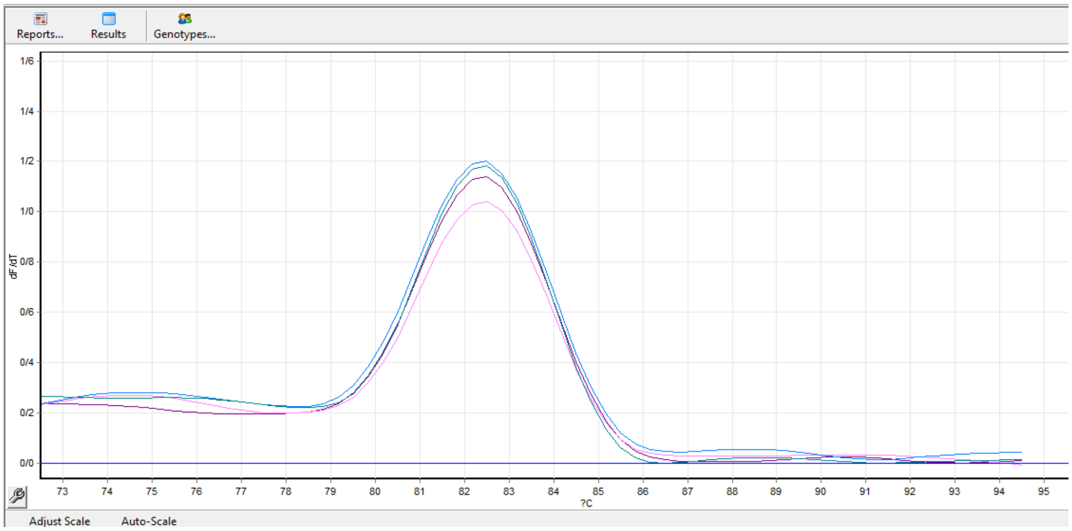

Figure 7: Screen shots from the Rotor-Gene 6000 software of the melting curve for TLR-4

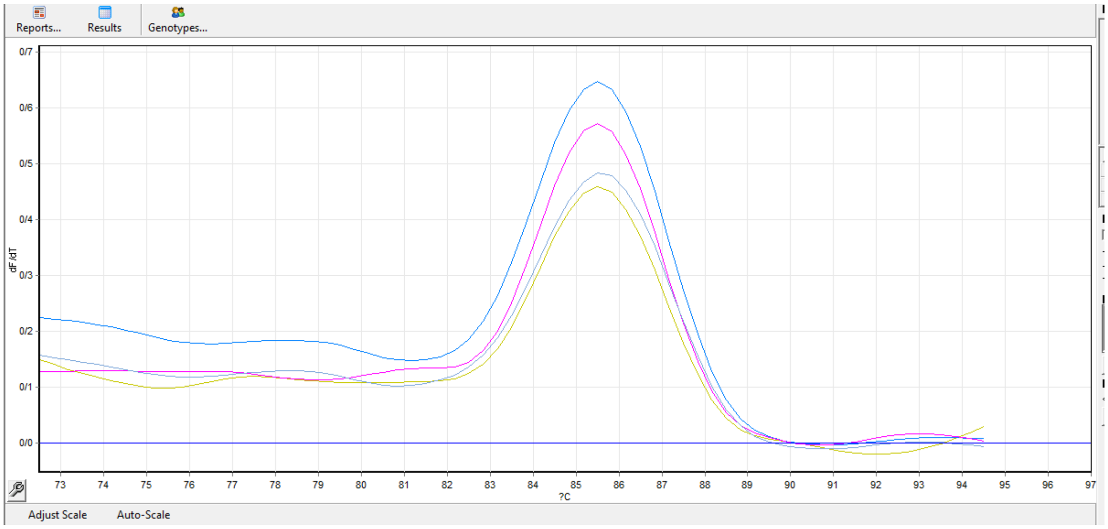

Figure 8: Screen shots from the Rotor-Gene 6000 software of the melting curve for TNF-α

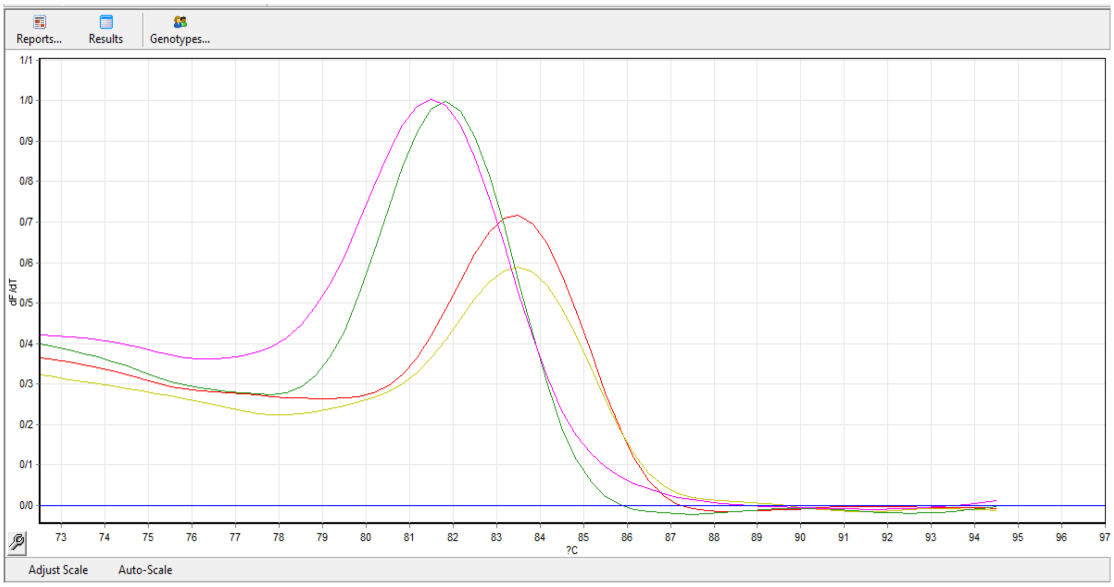

Figure 9: Screen shots from the Rotor-Gene 6000 software of the melting curve for mir-21 and mir-31

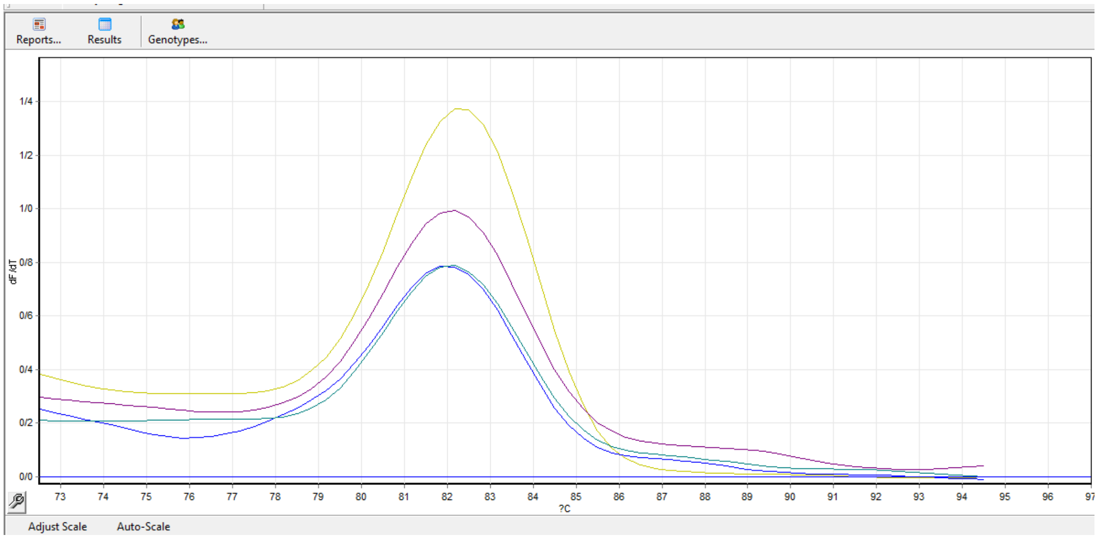

Figure 10: Screen shots from the Rotor-Gene 6000 software of the melting curve for SLCO

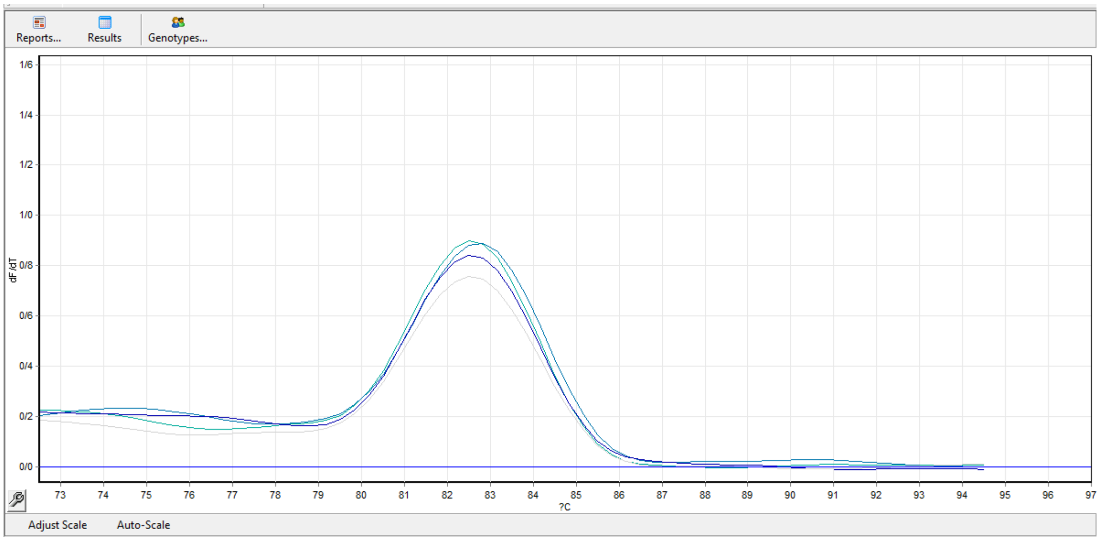

Figure 11: Screen shots from the Rotor-Gene 6000 software of the melting curve for U6
